# WEBCAMERA-BASED VISUAL PAIRED COMPARISON AS A REMOTE COGNITIVE SCREENING METHOD

**DOI:** 10.1093/geroni/igz038.1214

**Published:** 2019-11-08

**Authors:** Michelle Gray, Joshua Gills, Spencer A Smith, Emily Bates, Jordan M Glenn, Erica Madero, Nick Bott

**Affiliations:** Neurotrack, Redwood City, California, United States

## Abstract

Alzheimer’s disease (AD) is a form of dementia impacting memory and cognitive function of 131 million individuals worldwide. Though early cognitive decline detection is important, cognitive screening is limited among older adults and many cases go undetected. As easy-to-use cognitive assessments are not readily available to the general population, the purpose of this investigation was to determine the ability of a 5-minute webcamera-based eye-tracking cognitive assessment to discriminate between cognitively intact adults and adults with mild cognitive impairment (MCI) or AD. This prospective study included 56 participants (age=55.9±26.8) divided into three groups: younger cognitively intact (ages 18-46 years, n=25), older cognitively intact (ages >60 years, n=20), and older cognitively impaired participants with MCI or AD (ages>60 years, n=13). All participants completed the Digit Symbol Substitution Test (DSST) and Visual Paired Comparison test (VPC) to assess cognition. One-way ANOVA detected differences in cognition between groups. A Pearson correlation determined the association between cognitive assessments. Additionally, multiple regression determined the ability of VPC and age to predict DSST scores. Results revealed significant differences between cognitively intact and cognitively impaired groups for VPC (p=.001) and DSST (p<.001). Follow-up analyses revealed significant differences between cognitively intact and cognitively impaired adults (p=.005) with no differences between younger and older cognitively intact adults (p=.34). There was a significant association between the VPC and DSST cognitive assessments (r=.54, p<.001), with VPC and age accounting for 69% of the variation in DSST. These results support the use of webcamera-based VPC as a viable option when screening tool MCI/AD.

